# Exploring the Experiences of Pharmacy Students and Their Transition to Online Learning during COVID-19

**DOI:** 10.3390/pharmacy10050110

**Published:** 2022-09-02

**Authors:** Aleisha C Morling, Shou-Yu Wang, M. Joy Spark

**Affiliations:** 1 Discipline of Pharmacy, School of Rural Medicine, University of New England, Armidale, NSW 2351, Australia; 2 Discipline of Nursing, School of Health, University of New England, Armidale, NSW 2351, Australia

**Keywords:** online learning, pharmacy students, COVID-19, phenomenology, qualitative research

## Abstract

Due to the heavy focus on development of communication skills, compounding laboratories and many practical workshops, undertaking a registerable pharmacist qualification in an online format is typically not an option for students. COVID-19 presented on-campus pharmacy students with the opportunity to experience online learning. The aim of this study was to explore the experiences of on-campus pharmacy students who were required to move their studies to online learning during the COVID-19 pandemic. An interpretive phenomenological methodology was adopted, and semi-structured interviews were conducted with pharmacy students who were originally enrolled in on-campus learning and had to transition to online learning. Data were analyzed using a hermeneutic phenomenological approach whereby themes were identified to aid in the development of the phenomena guided by ‘lived experience’. Seven interviews were conducted with pharmacy students. Four emergent themes resulted from the interviews: (1) life as an on-campus pharmacy student, (2) preconceived ideas of online learning, (3) learning differences as an online pharmacy student and (4) the future of online pharmacy programs. Students were initially hesitant to transition to online learning due to preconceived ideas and expectations that may have tainted their overall experience. Pharmacy students preferred face-to-face learning due to their sociable personality and heavy dependence on peer and teacher support. All participants reported that they preferred face-to-face learning and acknowledged that fully online programs were not suited to their learning style or to the discipline of pharmacy. After their experience of online learning, participants believed that there was a place for online learning components in pharmacy courses. Lectures and some discussion workshops could be delivered online, but some aspects, such as compounding; dispensing; counselling; and demonstration of medication delivery devices, such as asthma inhalers and injectable diabetes products, should be delivered on campus.

## 1. Introduction

Pharmacy students, along with many scholars, were required to transition to online learning in 2020 to prevent the spread of the severe acute respiratory syndrome coronavirus 2 (SARS-CoV-2), during the global pandemic [1]. This provided on-campus students with the opportunity to experience online learning. Students had to change their routines based around their university studies and were also confronted with the many stressors related to viral disease outbreaks associated with the ongoing pandemic [2].

Traditionally, distance learning, also referred to as remote education, referred to study that was completed without the student and educator both being physically present in a traditional face-to-face learning environment [3,4]. A remote education program was offered either as fully remote or an amalgamation of distance and classroom teaching, often referred to as hybrid or blended learning [5,6]. Fully remote and blended higher education programs have been offered around the world by universities in response to an increase in student demand for increased flexibility with their studies [7]. Whereas distance education is not new to Australia, advancements in technology, such as computers and the internet, have revolutionized the way that students, teachers and their peers can communicate and manage their studies. Distance education has thus transitioned to online education or e-learning [4]; however, concerns about the effectiveness of learning via online education have instigated a growing plethora of ongoing research [8,9].

The efficacy and student perspectives of online learning have been investigated amongst higher educational students [5,8,10,11,12,13,14]. Prior to the COVID-19 pandemic, online learning was not usually an option for pharmacy students, so there are few reports about the experiences of pharmacy students with regard to online learning [3,9,15]. Only one Australian university offers an accredited course leading to a registerable pharmacist qualification as an online program [16]. This is a blended program, with students required to travel to the university for 2 to 6 days per enrolled unit to satisfy learning outcomes and skill development requirements. During the COVID-19 pandemic, many pharmacy programs had to develop a new approach to delivering learning materials, including consideration of accessing the internet, technologies and educational software [17,18]. Qualifications in pharmacy focus heavily on communication and laboratory skills, which are difficult to demonstrate via video conference or without specialized materials and equipment [19]. During the COVID-19 pandemic, pharmacy faculties were faced with the challenge of converting necessary practical skills to an online interpretation. Emergency remote teaching was adopted by Australian universities, with most electing to postpone assessments that could not be evaluated while fully online (e.g., extemporaneous compounding) [17,18]. Adaptions made to the pharmacy education program showed that changes to the course could be made to facilitate online learning, although the sustainability of these practices are yet to be explored.

Introducing a new learning style to students is complex. One of the challenges for higher education during the COVID-19 pandemic was changing traditional face-to-face learning to online learning in a short amount of time. Discipline leaders faced challenges and struggled with how to keep a holistic learning approach and ensure students engaged and adapted to the new learning style [20,21].

In addition, higher education students have been found to have mixed perceptions of online learning and be unsure of its efficacy in comparison to face-to-face delivery [2,10,11]. This may be explained by the barriers identified with respect to online learning. The main barrier reported to concern university students is the drive and motivation required to undertake self-directed learning [5]. Technology problems are also a concern expressed by university students, as these can hinder and slow the learning–teaching process [22,23]. Student learning preferences can also affect their perception of online learning. Prior to the pandemic, most (89%) pharmacy students had reported an interest in meeting for an in-person component when their lectures were delivered in an online format [15]. Many of these students identified the best way to deliver content as with live or recorded lectures. Face-to-face workshops continue to be considered beneficial to the learning process. Students also considered online platforms and technology to be essential to their academic success [3,7,9,15,23]. This highlights the shift in content delivery as technology becomes essential for a new generation of students progressing through tertiary education.

Investigation of pharmacy student online readiness, acceptability and satisfaction has been limited to quantitative studies using surveys [3,23,24]. There have been no reported qualitative studies that allowed pharmacy students to guide the discussion of their own experience of transitioning to online learning. There has been little to no investigation of how COVID-19 has changed the perceptions of online learning among pharmacy students. It is important to understand the perspectives of pharmacy students who have experienced both online and on-campus delivery of learning activities, as their feedback can influence the future delivery of their program of study and their academic performance [25,26]. A unique opportunity to explore the experiences of on-campus pharmacy students transitioning to online learning has been provided by the COVID-19 pandemic.

The aim of this study is to explore the effect that transitioning to online learning during the COVID-19 pandemic had on on-campus pharmacy students’ perspective of online learning and their learning experiences. Additional aims of the study include exploring the challenges and benefits of online education.

## 2. Method

An experience is best investigated with qualitative research methods [27]. Consequently, an interpretative phenomenological research design was adopted for this study to support the development of phenomena that arose from students’ lived experience [28,29]. Informed by the philosophy of Heidegger [30], the aim of this research design was to combine the researcher’s knowledge and experience with the data explored by participants without allowing personal bias to shadow understanding [31]. This study design allowed for a thorough understanding of students’ online learning experience and focused on revealing the hidden meanings in the accounts of the experience while considering the contexts of the participants [32,33]. In this qualitative research study, we used COREQ to report the findings [34]. Human research ethics approval was obtained from the Human Research Ethics Committee at the University of New England (Approval no. HE21-097).

The research team consisted of three researchers (A.C.M., S.Y.W. and M.J.S.), two of whom are experienced academics with doctorates. S.Y.W. has extensive practice in qualitative studies, and M.J.S. provided the insight of a pharmacy academic. S.Y.W., M.J.S. and A.C.M. were all involved with the data analysis process of this study.

Purposeful sampling was used to recruit pharmacy students located in the Australian states of Victoria and New South Wales. These two states together represent more than 50% of registered pharmacists in Australia [35] and 8 of the 18 universities with qualifications leading to registration as a pharmacist [16]. An email was sent to each pharmacy department lead in both states (8 universities) using contact information that was publicly available from the university websites. The department lead was asked to distribute a study invitation via email to their current pharmacy cohort on behalf of the researchers. Interested participants made direct contact with the researcher (A.M.) to ensure that they met the required research criteria (see Table 1) prior to organizing an interview.

Semi-structured interviews were conducted with open-ended questions to allow participants to reflect on their own online learning experience [36]. A semi-structured interview guide (Appendix A) was followed to direct the discussion and was piloted with test interviews prior to official data collection. Individual interviews allowed for an in-depth analysis and a higher potential for insight into hidden and complex topics [37]. Interviews were audio-recorded via the online conferencing platform Zoom and transcribed verbatim. Participants gave verbal, recorded consent before reflecting on and sharing their experiences of learning on campus and comparing their experience to their online learning journey. Data collection continued until data saturation was reached, i.e., new information did not provide further insight into the emerging themes [38]. A reflective entry was written after each interview to document subjective aspects, which would later assist with ongoing analysis and reflexivity. All electronic data, such as audio recordings and interview transcripts, are securely stored on the University of New England’s centrally managed cloud server and will be kept for a minimum duration of 5 years.

Data analysis began after the first interview and continued throughout the data collection period. Interviews were analyzed manually by the researchers using the hermeneutic phenomenological approach to analysis [39]. This involved a continuous, reflexive process whereby themes emerge, and the researchers returned to the data to further understand and interpret the meaning [31,40]. The audio recordings and transcripts of each interview were reviewed numerous times to allow the researchers to become immersed in the experience described by the students. The emotion associated with responses, as well as non-verbal cues, were taken into consideration. Phrases regarded to be of importance by evoking further thinking and interest were documented, and each interview was continually revisited for clarification and understanding. Emerging themes and their connections were explored, and a description of the phenomena came to fruition, supported and guided by the “lived experience” of the participant.

## 3. Results

A total of seven interviews were conducted with students from five universities. Interviews varied from 30 to 60 min in length. After five interviews, data began to repeat, without the evolution of any new themes or insight into the phenomena. At this point, another two interviews were conducted to ascertain whether the phenomena framed by lived experience had been captured. The age of participants ranged from 21 to 33 years and included four female and three male students (see Table 2). Four of the seven participants started their pharmacist qualification immediately after high school, and the other participants began a registerable pharmacist qualification as a mature-aged student. No participants had any experience with online learning prior to the COVID-19 pandemic. Interviews were held between June and August 2021, and all participants had completed at least two teaching periods of online learning. Participants are identified as Participant 1, Participant 2, etc., to protect their identity and uphold confidentiality. The analysis of the interviews revealed four major themes: (1) life as an on-campus pharmacy student, (2) preconceived ideas of online learning; (3) learning differences as an online pharmacy student characterized by communication difficulties, lack of motivation, the intimidation of online environments and learning flexibility; and (4) the future of online pharmacy programs.

### 3.1. Life as an on-Campus Pharmacy Student

Participating students reflected fondly on their time on campus. Being surrounded by like-minded students provided a sense of community unique to on-campus learning. “It becomes your second home, and you feel a sense of belonging there to a place and to a cohort of people” [Participant 4]. Having all pharmacy students together in a common on-campus learning environment made it easy to establish friendship and support groups. It facilitated organic discussion, whereby students were able to thrive as a result of sharing ideas with one another. These social connections were highly valued by all participants.

*“I think it’s obvious that I thrive off having people around me. I think I learn a lot better. I like to bounce ideas off people around me and run through questions and scenarios and bounce ideas off people that typically you’re only going to be comfortable with if you have a bit of a relationship with them”*

—Participant 7.

*“You’re in a class on campus and you have students everywhere who are easy to talk to. If you need a hand, people aren’t shy to ask questions, people aren’t shy to introduce themselves, especially when you’re meeting them for the first time. People want to make friends while they’re at uni”*

—Participant 1.

Pharmacy students’ experiences of being on campus also allowed for easy and timely access to teachers when needed. Establishing a rapport with the pharmacy academics also made it easier to approach the staff for help and to ask questions. “It’s very easy to get questions answered and seek help if needed”—Participant 2.

*“Being a very small cohort, we did have a lot of contact with our tutors. They were very specific to pharmacy, which is really good, and we did have that really close relationship with a lot of them. We’d see them day in and day out when we were on campus. So, you sort of got to know them, they all knew us by name”*

—Participant 5.

The on-campus lifestyle allowed for a successful learning environment, away from home and any other distractions. The ability to separate home and work from the learning environment was key to facilitating the appropriate mindset required for study, completing assignments and concentration.

*“I don’t study from home. Home is home. University is at university, to the point I would go in at night-time to use the library and stuff. Even though I could use my desk at home, I wanted to be in that space that I associated with doing study”*

—Participant 3.

### 3.2. Preconceived Ideas of Online Learning

Exploring participants’ opinions of online learning prior to their own experience highlighted a lack of online course understanding and awareness. Most participants were unaware that an online/distance pharmacy course is offered in Australia and had never considered nor believed it an option for obtaining a registerable pharmacist qualification. Only one participant had contemplated undertaking an online certificate prior to their own online experience, whereas others had never contemplated the alternative learning approach.

*“It wasn’t anything I ever considered nor was interested in. I think I just felt it was for rural students. I think I felt it was for people perhaps living overseas at the time. That was my perception of online learning and also for people who work and had to be flexible for their school hours”*

—Participant 4.

*“My idea of it was that you were given the task and you just do it when you get a chance almost. And there was very limited interaction with other people. It was just like you watch these videos or you do this quiz and that’s your learning”*

—Participant 5.

Each participant expressed a hesitancy and uncertainty about online learning. Terrified, scared, devastated, unsure and anxious were the main words and emotions expressed by students to explain how they reacted to moving to an online learning platform. The level of uncertainty and concern ultimately began to negatively impact the mental health of some students. “I was terrified that I would fail university immediately. I was so scared, and it started affecting my ability to go to sleep at night-time” [Participant 3].

*“I was quite devastated in the beginning. I don’t think anybody likes change. I’ll admit I don’t. I was very comfortable with my school and life balance. I lost a lot of sleep over it in the beginning, wondering how this could possibly work”*

—Participant 4.

One participant also shared their concern about the quality of online teaching.

*“I did subtly in the back of my mind think it was not as beneficial or it was not as high standard as face-to-face learning… I’m so used to doing face-to-face learning throughout my first two years at university and through all my high school, my primary school. I just always thought that it was a superior method of teaching and just never really considered that online was ever really a viable learning option”*

—Participant 2.

### 3.3. Learning Differences as an Online Pharmacy Student

During the interviews, participants were continually asked to reflect upon the differences between on-campus and online learning. This comparison of delivery methods evolved into a discussion about the challenges and benefits associated with online education. Four main subthemes were identified by the participants.

#### 3.3.1. Communication Challenges

Participants found it difficult to ask questions of their teachers and receive responses in real time. They found that online lectures and workshops dedicated little time to asking questions and seeking assistance. Generally, participants reported resorting to email to communicate with teachers. This communication delay encouraged participants to search elsewhere for answers to prevent interruption of their ongoing study routine.

*“When I needed to email, I really wanted an immediate response like I would on a face-to-face visit, and I didn’t get that. It could sometimes be two days before I got an answer and that of course held up my work. And that is the crux of my frustration with online study”*

—Participant 4.

*“We’d have perhaps 10 min of question time and sometimes your questions didn’t come till after the lecture. So yeah, it was difficult. You couldn’t ask the teacher the following day at school because you weren’t there”*

—Participant 3.

Participants also identified online communication to be missing a humanized connection, describing interactions as impersonal. Multiple participants acknowledged the difficulty of recognizing social cues over online platforms, especially during assessments involving interactions with mock patients.

*“Online you’re talking to someone through for a screen and it’s not the same [as being in-person]. You don’t really know what their body language is, you can’t really like interpret what they’re thinking. If you’re looking at someone through a screen, whereas when you’re in person with someone you can kind of feel the energy, just trying to understand better how they’re feeling”*

—Participant 1.

#### 3.3.2. Lack of Motivation

After moving to online learning, participants found their motivation to study deteriorated, which also affected their academic performance. This was attributed to the loss of structure to their education schedule, distractions associated with their learning environment and the loss of interaction with peers and teachers. “Even just on an academic scale, I could see myself doing much better on campus than now online, I was doing better. My grades were much higher while I was on campus rather than online” [Participant 1]. However, one participant shared that the absence of social distractions allowed them more time to study.

*“So that was year three, 2020, it’s been my best year so far. So, in terms of results and things like that, I feel like I was able to adjust well enough and be able to learn the content and do well on the exams and come out with good marks”*

—Participant 3.

Participants struggled with the loss of structure to their learning schedule, as they were previously familiar with set weekly lectures and workshop times. There was a loss of personal routine, as there were no expectations to be on campus at a given time.

*“Sometimes there’s too much freedom. I like the rigidity of you be here at this time and we’re going to do this content. And when I was sort of left off in the, here it is uploaded, watch it in your time, I was quite relaxed about it and often skipped recorded lectures”*

—Participant 3.

The inability to separate the home and learning environment was a major hinderance to some participants’ motivation. Distractions present around the home, such as family and the pressures of everyday tasks (cooking, cleaning, etc.), prevented the appropriate mindset required for learning and concentrating. On campus, participants rely heavily on their peers and teachers to facilitate a successful learning environment. “I think on campus, my teachers and my peers motivated me and at home I have to motivate myself. And there’s a huge difference there” [Participant 4].

*“It’s just that mindset that you’re sitting down and you’re looking at a computer screen for however many hours and it’s just like, you’re mentally not there. Whereas when you’re sitting down and you’re face-to-face with students all around, you know, you feel more motivated”*

—Participant 1.

#### 3.3.3. Intimidation of Online Environments

The participants who started university immediately after secondary school revealed that speaking in an online platform was daunting. Those who struggled to contribute in a face-to-face group environment found speaking in front of peers in an online setting to be more confronting. “A lot of people don’t realize how intimidating it could be for like a student to speak up through live online classes… It’s hard, people are shy to communicate online” [Participant 1].

*“I do feel like [student contribution] dropped once we went online. There was a lot more interaction when we were there on campus… people were very much more reserved when they were online. I feel a lot of the teachers were met with more silence than what they were used to”*

—Participant 5.

One participant overcame this challenge by turning their camera off so their face was hidden should they answer a question incorrectly. “I was a lot more confident to turn my microphone on and give answers because you don’t have that ridicule or embarrassment if you get it wrong, no one can see your face” [Participant 2]. However, the mature-aged participants showed no hesitation to contribute to online discussions. When asked about their own self-confidence, they were invested in making the most of the limited time they had with their teachers.

*“I didn’t care if anyone thought I was dumb, it’s my education, it’s my degree. I’m going to make this work for me. I think that just comes with a bit of maturity and time”*

—Participant 3.

#### 3.3.4. Learning Flexibility

Participants were quick to highlight the barriers that they associated with their online learning journey, but all mentioned the advantage of learning flexibility when prompted. Without any travel time to and from university to allow for, participants had suddenly gained many free hours during the week. “I spent two hours a day travelling, and I gained that with online study, that was huge. And it was spent travelling in the car or on public transport” [Participant 4]. The ability to manage their learning in their own time gave participants the freedom to better manage other aspects of their life. This included accomplishing a healthier work/life balance and having more time to spend with family.

*“One of my friends, she’s a single mum with a five-year-old and she actually doesn’t even come to class. She’s like, I get way more of it just popping in my headphones and watching the recordings. So that just suits her, and she’s made that work for her”*

—Participant 3.

*“I think it’s really good to be able to do these things in your own time, if you’re somebody that has a lot of other responsibilities as well… When I was working, I was able to do study after work and things like that, so I found that really helpful”*

—Participant 5.

### 3.4. The Future of Online Pharmacy Programs

All participants in this study strongly expressed that they preferred face-to-face delivery over online delivery. Many participants agreed that the quality of learning provided online was different but still comparable to learning face-to-face. Some believed that there was a place for online learning components in pharmacy courses, such as for the delivery of lectures, but there were some aspects that had to be delivered on campus. This included compounding, dispensing, counselling and the demonstration of medication delivery devices, such as asthma inhalers and injectable diabetes products.

*“You lose that hands on experience and especially for the asthma puffers and the diabetes training, you know, we sort of missed out on that… You get that deeper understanding from having physically touched it and used it”*

—Participant 3.

The majority of participants were opposed to the progression to entirely online courses for any future pharmacy programs. “I can’t see any benefit of continuing online learning as we are” [Participant 2].

*“I hope [online pharmacy courses] wouldn’t continue. I feel like you’re going to miss out so much, especially learning to talk to people face-to-face. Yeah, we have telehealth, but we’re not there with pharmacy yet… Lots of people I feel like will very much struggle”*

—Participant 6.

When asked if they would consider undertaking other online units in the future, the discussion of adaptation and acceptance arose.

*“I fought against it in the beginning, but now I am fine and now I would happily engage in online learning again. It wouldn’t be my first preference. I will always prefer to do on-campus learning, but if that’s not available to me, I wouldn’t be too shy to try online learning again”*

—Participant 4.

*“After sort of getting used to it, I actually did find it was very, it wasn’t as difficult as what I had sort of initially thought… As long as you’re keeping on top of things, you’re planning ahead really well. I think it’s, that’d be something that I’d be willing to try”*

—Participant 5.

## 4. Discussion

In the present study, we conducted an in-depth exploration into the learning journey undertaken by pharmacy students during the COVID-19 pandemic. The study afforded the opportunity for pharmacy students to discuss aspects of the change from on-campus to online learning that substantially impacted their experience. The findings show that participants were initially reluctant to transition from on-campus university life to online learning. Concerns about the quality, lack of communication and support from their university were expressed. Participating pharmacy students were quick to identify major barriers to online learning, such as communication challenges, lack of motivation and social intimidation in online environments. The sole advantage recognized was an increase in learning flexibility, and all participants agreed that they preferred face-to-face learning over online learning. Participants believed that there is a place for online learning components in pharmacy courses, such as the online delivery of lectures, and that there are some aspects that have to be delivered on campus. These include compounding, dispensing, counselling and the demonstration of medication delivery devices, such as asthma inhalers and injectable diabetes products.

A change in learning style can be difficult for any student, especially with the many additional stressors related to lockdowns associated with the COVID-19 pandemic. From as young as 5 years old, most children attend education in a traditional classroom design that continues through to secondary school [41]. Consequently, it was not surprising that pharmacy students were initially hesitant about the learning change associated with the global pandemic. Students had to transition unexpectedly to a style of learning that most had never considered.

The participants assumed that online education existed only for those living in rural areas, working full-time jobs or for courses with few practical components. The lack of awareness of online course availability and the misconception of whom online courses are intended and designed for is echoed by other higher education students [13]. Expectations of online learning are formed by a student’s previous experience and attitude toward traditional classroom learning. As all participants had no prior experience with online learning, their preconceptions of this learning style were based on perceived preference and their personalities [12]. Their preconception consequently influenced their experience and perceptions of their online journey. Participants experienced online learning as more difficult because it required a higher level of self-motivation, dedication and learning independence. These personal attributes were associated with the participants’ ability and willingness to accept and adapt to the learning changes. Willingness and receptibility to change have been shown to be influenced by an individual’s personality [42]. Resistance to change may lead to learning dissatisfaction and complications if the individual’s personality is not conducive to independent and self-directed models of learning. Pharmacy is a university degree heavily focused on an outcome of effective communication, and this may naturally attract a social personality [43]. These participants may have found it harder to move to online study due to their natural sociable personality and heavy dependence on peer support. The social support of a university degree was of considerable importance to participants, including strong support relationships with staff resulting from smaller discipline cohorts in comparison to other courses [44].

A blend of traditional classroom and online learning methods were preferred by most participants. This is consistent with findings from studies conducted prior to COVID-19, wherein pharmacy students shared their preference for the extent of the inclusion of technology in their learning [24]. Blended learning provides students with flexible learning opportunities while still incorporating important face-to-face time with educators. There is a higher level of student engagement associated with blended learning when compared to fully online learning. The increase in engagement provides students with a sense of community that has been shown to have a positive impact on academic performance [45].

There were challenges associated with online learning. Communication delay, a lack of motivation to study and the intimidation of online environments were among the wide-ranging challenges to online learning identified and could apply to many tertiary education students. This is supported by similar outcomes being reported after the experiences of other higher education students were explored [46,47]. Participants also had a heightened concern for the underdevelopment of communication skills and practical knowledge specific to pharmacy practice. The effectiveness of online simulated patient interactions was questioned due to the difficulty of judging social cues and establishing a rapport with the patient. This reflects the consensus of medical students, who prefer face-to-face patient interactions, although online simulations have been shown to be an effective alternative [48].

### 4.1. Implications for Practice and Future Research

This study provides insight into the preferred blended learning format for pharmacy students. It also provides guidance for pharmacy academics as universities prepare for the future of learning post COVID-19. Should universities begin to permanently offer a blended learning approach for pharmacy programs, they need to consider the importance of peer and teacher relationships. This has been shown to drive academic outcomes and, ultimately, the satisfaction of students’ university learning journey [49]. The findings indicate a further need for research to determine the most effective learning delivery method to balance student satisfaction and the successful development of key communication and practical skills essential for pharmacists.

### 4.2. Limitations

The small number of interviews could be considered a limitation; however, it was within the range of 5 to 12 interviews previously reported to be sufficient to reach data saturation [40,50]. All interviews only included Victorian or New South Wales pharmacy students, so may not be representative of the views of those from other states or countries. Our results may also be subject to volunteer bias, whereby those willing to participate might be more likely to have a sociable or outgoing personality. These individuals typically thrive on the social nature associated with face-to-face environments, such as on-campus learning. This may mean that this study is missing the exploration of pharmacy students with opposing views to the results reported herein.

## 5. Conclusions

Adapting to a new learning style is a complex journey. Participants were initially hesitant to transition to online learning due to their negative expectations influenced by their sociable personality and prior experience with traditional classroom teaching. Flexibility was reported as a benefit of online learning. The identified challenges of online learning included communication difficulties, lack of motivation and the intimidation of online environments. There was also concern about the potential for underdeveloped communication skills associated with online delivery platforms. All participants reported that they preferred face-to-face learning and acknowledged that fully online programs were not suited to their learning style or to the discipline of pharmacy. After their experience of online learning, participants believed that there was a place for online learning components in pharmacy courses. Lectures and some discussion workshops could be delivered online, but some aspects, such as compounding, dispensing, counselling and the demonstration of medication delivery devices, such as asthma inhalers and injectable diabetes products, have to be delivered on campus.

## Figures and Tables

**Table 1:** Inclusion criteria for subject participation.

Inclusion Criteria
Older than 18 years old Previously an on-campus student Able to interview in English Agree to participate in recorded interview Undertaken ≥1 online university unit Enrolled in Pharmacy Board of Australia-approved pharmacy program

**Table 2:** Demographic data of participants (*n* = 7).

Participant	Age	Gender	Year of Study in 2020	University (State)
1	21	Female	Second	A (Vic)
2	22	Male	Third	A (Vic)
3	31	Male	Third	B (NSW)
4	33	Female	Third	C (NSW)
5	23	Female	Fourth	A (Vic)
6	22	Female	Third	D (NSW)
7	25	Male	Second	E (Vic)

## Data Availability

The data presented in this study are openly available in Research UNE at [doi:10.25952/ndyv-w562], reference number [https://hdl.handle.net/1959.11/53268] (accessed on 16 August 2022).

## Appendix A. Semi-Structured Interview Guide

(1) Tell me about your learning experience as an on-campus student.
  Why did you choose on-campus learning in the first place?
(2) What were your perceptions of online learning prior to COVID-19?
  What is your definition of online learning?
  What do you think influenced these perceptions of online learning?
  Have you ever considered taking an online course before? Why?
(3) Tell me about your online learning experience during COVID-19.
  How did you feel when you found out you would be doing online study?
  What were the main barriers you identified to online learning?
  What were the benefits of online learning?
(4) How did your views of online study change as the year progressed?
  What do you think influenced this view about online learning?
(5) How has your approach to learning or study techniques changed?
(6) Do you think pharmacy programs can progress as an online alternative? Why?
  What would you say are the main characteristics of someone who would do well in an online learning environment?
(7) Would you consider taking online units in future study? Why?
(8) What advice would you give to a pharmacy student who is taking an online course for the first time?
(9) What suggestions would you give to pharmacy staff members teaching online courses?
  How do you think your university handled the change from on-campus learning to online learning?
(10) Is there any other additional information you would like to discuss regarding your experience of online learning?
(11) Is there anything you would like to add any of the previous questions we have explored today?
